# Biochemical characterisation of LigN, an NAD^+^-dependent DNA ligase from the halophilic euryarchaeon *Haloferax volcanii *that displays maximal *in vitro *activity at high salt concentrations

**DOI:** 10.1186/1471-2199-7-44

**Published:** 2006-11-28

**Authors:** Laetitia Poidevin, Stuart A MacNeill

**Affiliations:** 1 Institute of Molecular Biology and Physiology, University of Copenhagen, Sølvgade 83H, 1307 Copenhagen, Denmark; 2 From January 1^st ^2007: Københavns Biocenter, University of Copenhagen, Ole Maaløes Vej 5, 2100 Copenhagen ∅, Denmark

## Abstract

**Background:**

DNA ligases are required for DNA strand joining in all forms of cellular life. NAD^+^-dependent DNA ligases are found primarily in eubacteria but also in some eukaryotic viruses, bacteriophage and archaea. Among the archaeal NAD^+^-dependent DNA ligases is the LigN enzyme of the halophilic euryarchaeon *Haloferax volcanii*, the gene for which was apparently acquired by *Hfx.volcanii *through lateral gene transfer (LGT) from a halophilic eubacterium. Genetic studies show that the LGT-acquired LigN enzyme shares an essential function with the native *Hfx.volcanii *ATP-dependent DNA ligase protein LigA.

**Results:**

To characterise the enzymatic properties of the LigN protein, wild-type and three mutant forms of the LigN protein were separately expressed in recombinant form in *E.coli *and purified to apparent homogeneity by immobilised metal ion affinity chromatography (IMAC). Non-isotopic DNA ligase activity assays using λ DNA restriction fragments with 12 bp *cos *cohesive ends were used to show that LigN activity was dependent on addition of divalent cations and salt. No activity was detected in the absence of KCl, whereas maximum activity could be detected at 3.2 M KCl, close to the intracellular KCl concentration of *Hfx.volcanii *cells.

**Conclusion:**

LigN is unique amongst characterised DNA ligase enzymes in displaying maximal DNA strand joining activity at high (> 3 M) salt levels. As such the LigN enzyme has potential both as a novel tool for biotechnology and as a model enzyme for studying the adaptation of proteins to high intracellular salt levels.

## Background

DNA ligases play key roles in all forms of cellular life [1]. Two families of DNA ligase, differing in their cofactor specificity, are characteristic of the two major branches of evolution, the eubacteria and the eukarya/archaea. NAD^+^-dependent DNA ligases (EC 6.5.1.2) are encoded predominantly by eubacteria [2] but also by certain eukaryotic viruses, such as the entomopoxviruses [3] and mimiviruses [4], and by some bacteriophage [5,6]. In contrast, ATP-dependent DNA ligases (EC 6.5.1.1) are characteristic of eukaryotic and archaeal cells but are also found encoded by certain eukaryotic viruses, bacteriophage and eubacteria [7]. The mechanism of DNA ligation is similar for both types of enzyme [1]. In the first step, attack on the α-phosphorus of either NAD^+ ^or ATP by the enzyme results in formation of an enzyme-adenylate intermediate (AMP is covalently linked to a lysine residue) and release of either NMP or pyrophosphate. In the second step, the AMP moiety is transferred to the 5' end of the nicked DNA strand to form a DNA-adenylate complex. Finally, attack by the 3'OH group of the nicked DNA strand on the DNA-adenylate, catalysed by the enzyme, results in DNA strand joining and AMP release [1].

**Figure 1:**
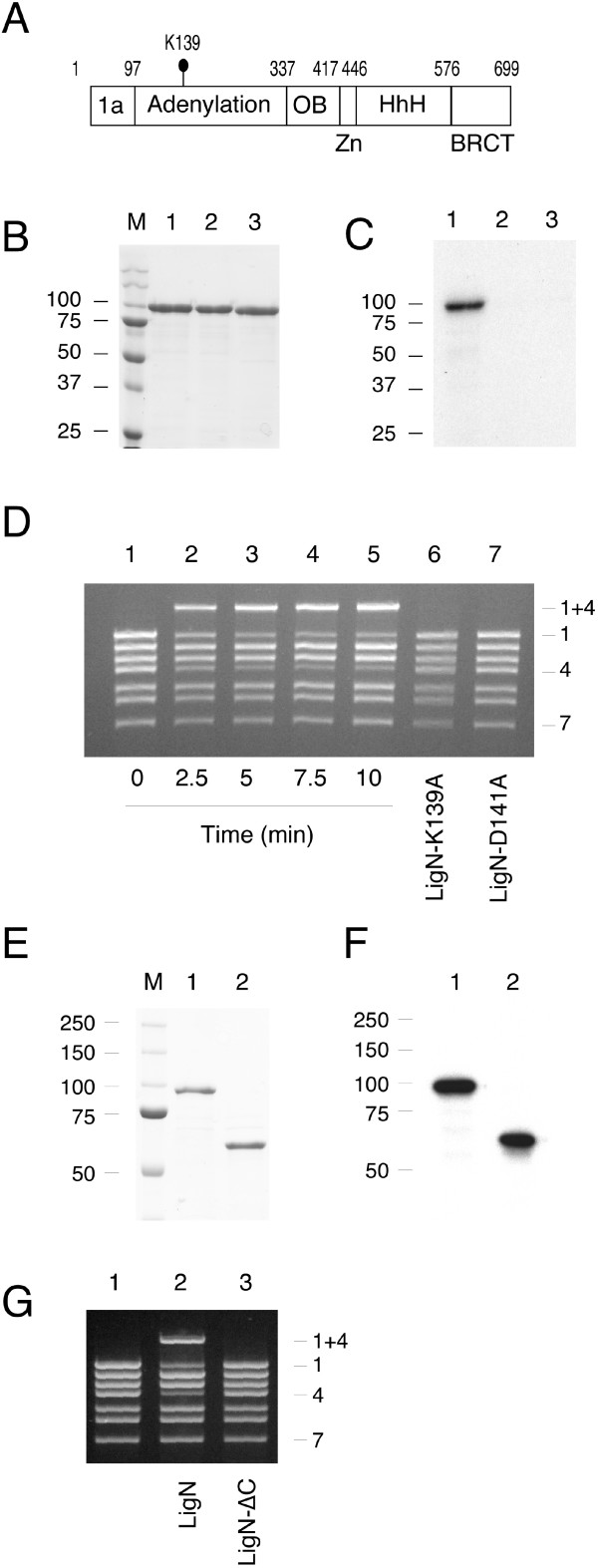
**Recombinant LigN is active as a DNA ligase**. A. Schematic representation of *Hfx.volcanii *LigN protein structure showing the six structural domains that characterise this family of enzymes. B. Purified LigN, LigN-K139A and LigN-D141A proteins (lanes 1, 2 and 3 respectively) visualised by PAGE-Blue G90 staining following 10% SDS-PAGE. 5 μg of each protein was loaded per lane. The lane marked M contains molecular weight markers with molecular weights (in kDa) shown to the left. C. Adenylation assay. Purified LigN, LigN-K139A and LigN-D141A proteins (lanes 1, 2 and 3 respectively) were incubated with [^32^P]NAD^+^, as described in the Methods, before being subjected to SDS-PAGE. Adenylated proteins were detected by autoradiography of the dried gel. D. Ligase activity assays carried out using λ *Bst*EII-digested DNA, as described in the Methods. Reactions were performed in 2.5 M KCl at 45°C with no added enzyme (lane 1, 10 minute incubation), LigN (lanes 2 – 5, incubation for 2.5, 5, 7.5 and 10 minutes), LigN-K139A (lane 6, 10 minute incubation) and LigN-K141A (lane 7, 10 minute incubation). E. Purified LigN and LigN-ΔC visualised by PAGE-Blue G90 staining following 10% SDS-PAGE. 5 μg of each protein was loaded per lane. The lane marked M contains molecular weight markers with molecular weights (in kDa) shown to the left. F. Adenylation assay. Purified LigN (lane 1) and LigN-ΔC (lane 2) were incubated with [^32^P]NAD^+^, as described in the Methods, before being subjected to SDS-PAGE. Adenylated proteins were detected by autoradiography of the dried gel. G. DNA ligase activity assays performed as described in Methods without added enzyme (lane 1) or using LigN (lane 2) or LigN-ΔC (lane 3). LigN-ΔC was inactive in the assay.

The euryarchaeal organism *Haloferax volcanii *is a halophile that was originally isolated in samples taken from the Dead Sea [8]. The organism grows aerobically with an optimal temperature of 45°C in medium containing 20% NaCl. Previously, this organism was shown to encode both ATP- and NAD^+^-dependent DNA ligase enzymes, designated LigA and LigN respectively [9]. Protein sequence analysis indicated that the gene encoding the LigN enzyme had been acquired by LGT from a eubacterium. At the sequence level, LigN closely resembles an archetypal eubacterial NAD^+^-dependent ligase enzyme [10]: it possesses a core ligase domain that is made up of adenylation and OB-fold sub-domains and which is flanked on the N-terminal side by a putative NAD^+^-recognition domain (domain 1a) and on the C-terminal side by a C4 zinc finger motif, a helix-hairpin-helix (HhH) domain and a BRCT domain (see Figure 1A). Genetic analysis showed that neither of the genes encoding the LigA or LigN enzymes is essential for *Hfx.volcanii *cell viability, but simultaneous deletion of both is lethal, indicating that the two enzymes, native and acquired, share at least one essential function, presumably the ligation of Okazaki fragments during chromosomal DNA replication [9].

Two different strategies allow halophilic organisms to cope in high salt environments [11,12]. The most widespread, seen in most aerobic halophilic eubacteria as well as in halophilic archaeal methanogens, involves the synthesis or accumulation of high intracellular levels of low molecular weight organic osmotic solutes, so-called *compatible solutes*, such as trehalose, betaine or proline [11,12]. Intracellular salt levels are kept to a minimum by active pumping of ions out of cells, so ensuring that the cells' enzymes, which are not salt-adapted, can function normally.

The alternative strategy is to accumulate high intracellular concentrations of KCl, to balance the concentrations of NaCl and other salts present in the extracellular environment [11,12]. This strategy is used by halophilic archaea of the order *Halobacteriales*, such as *Hfx.volcanii*, *Halobacterium salinarum *and *Haloarcula marismortui*, as well as eubacteria such as *Salinibacter ruber *[13-15] or members of the anaerobic order *Haloanaerobiales *[16]. In the case of *Hfx.volcanii*, the intracellular KCl concentration has been measured as 3.6 M [17]. Not surprisingly, the presence of such high intercellular salt levels has greatly influenced protein sequence and enzyme mechanism in these organisms. One such adaptation is reflected in the amino acid composition of proteins expressed in halophilic archaea. These are enriched in the acidic amino acids, glutamic acid and aspartic acid, and are reduced in basic and hydrophobic amino acids. These modifications are presumably required to maintain protein solubility, stability and function in high salt. Likely mechanisms for this have been discussed elsewhere [18].

Here we report biochemical analysis of the properties of the NAD^+^-dependent DNA ligase LigN from *Hfx.volcanii*. Using a non-isotopic DNA ligase assay based on λ DNA [19] we show that LigN enzyme has no activity in the absence of added KCl and increasing activity up to KCl concentrations greater than 3 M. LigN is the first DNA ligase enzyme to be characterised with such extreme salt tolerance.

## Results

### Expression and purification of recombinant LigN proteins

*Hfx.volcanii *LigN was expressed in *E.coli *from the kanamycin-resistant plasmid pET47b-HfxLigN as described previously [9]. To facilitate protein purification, the recombinant LigN protein carries an N-terminal hexahistidine tag as part of a 20 amino acid leader sequence that also contains a cleavage site for the HRV 3C protease, although the latter was not used in this study. Two additional expression vectors were constructed by introducing mutations into the LigN coding region by PCR overlap extension mutagenesis (see Methods). The resulting proteins differed from the wild-type in having either lysine 139 (mutant LigN-K139A) or aspartate 141 (mutant LigN-D141A) replaced with alanine. These amino acids are located within the adenylation domain of the enzyme in conserved ligase motif I (Figure 1A).

Protein expression was performed in *E.coli *Rosetta 2 (DE3) [pLysS]. This strain carries the pACYC184-derived chloramphenicol-resistant plasmid pRARE2 that harbors genes encoding tRNAs for the codons AUA, AGG, AGA, CUA, CCC, GGA and CGG, to enhance translation of non-native proteins where this would otherwise be limited by the codon usage of *E.coli *(note that the *ligN*^+ ^ORF has a GC content of 70%), as well as genes encoding T7 lysozyme and the *lac *repressor. The strain is also lysogenic for the λ DE3 prophage that contains the T7 RNA polymerase gene under the control of the IPTG-inducible *lac*UV5 promoter (see Methods).

Cultures were induced with 1 mM IPTG at 37°C for 4 hours. All three proteins were expressed in soluble form and could be purified to apparent homogeneity by Ni-NTA affinity chromatography in buffers containing 2 M KCl. Figure 1B shows the purified proteins. All three proteins migrate with an apparent molecular weight of ~100 kDa, significantly slower than would be expected from the predicted molecular weight of the proteins (75.8 kDa). This property is shared by many halophilic proteins and has been attributed to the high charge on the protein surface [12]. Slight differences in the mobility of the three proteins in relation to one another (see Figure 1B) can be attributed to the K139A and D141A amino acid substitutions and perhaps also to adenylation state of the recombinant proteins (see below).

### Adenylation assay

As described above, the first step of the ligation reaction requires that the ligase enzyme becomes adenylated to form a covalent enzyme-adenylate intermediate [2]. Adenylation takes place on a conserved lysine residue in the motif I sequence KxDG. In the *Hfx.volcanii *LigN sequence, this crucial catalytic residue is lysine 139. In other NAD^+^-dependent DNA ligases, the aspartate residue in motif I (equivalent to aspartate 141 in LigN) is generally not required for adenylation but is required for subsequent steps [2].

Previously we showed that the recombinant LigN protein could be adenylated *in vitro *by [^32^P]NAD^+ ^but not [α-^32^P]ATP [9]. Under the conditions used, approximately 65% of the LigN molecules became adenylated in the *in vitro *assay (data not shown). To examine the roles of lysine 139 and aspartate 141, we tested the ability of the two mutant proteins, LigN-K139A and LigN-D141A to be adenylated by [^32^P]NAD^+^. We found that both mutations reduced incorporation of radiolabel into LigN by > 99% (Figure 1C and data not shown, see also Discussion).

### Non-isotopic DNA ligase assay

To assay the ligation activity of the recombinant LigN protein, bacteriophage λ DNA digested with the restriction endonuclease *Bst*EII was used as a substrate in assays performed according to the method developed by Muerhoff and colleagues to measure the activity of the NAD^+^-dependent ligase from *Thermus thermophilus *[19]. Digestion of λ DNA with *Bst*EII produces fourteen DNA fragments ranging in size from 702 to 8454 bp. Fragments 1 and 4 (8454 and 5686 bp respectively) have 12 bp cohesive ends that are stably annealed and can therefore act as the substrate in a ligation reaction performed at 45°C, the optimal growth temperature of *Hfx.volcanii*. Following the ligation reaction, separation of the ligated 14 kb product from non-ligated substrate fragments is accomplished by ethidium bromide staining and agarose gel electrophoresis [19]. Imaging software is used to quantify the amount of product formed (see Methods).

Initially, the activity of the recombinant LigN protein was assayed at 45°C under conditions optimised for the commercially-available NAD^+^-dependent ligase from *Thermus aquaticus *(*Taq *DNA ligase) but supplemented by addition of KCl to a final concentration of 2.5 M. Figure 1D shows the results of this assay. LigN activity was readily detectable, with the product quantity reaching a maximum by 7.5 minutes (Figure 1D, lanes 1–5). Under these conditions, ~90% of the input substrate DNA was converted to product; longer incubations periods did not result in an increase in product amount (data not shown). As predicted, no activity could be detected with either of the adenylation-defective mutants LigN-K139A or LigN-D141A (Figure 1D, lanes 6 and 7 respectively). This is the first time that ligase activity has been demonstrated for the LigN protein and the first time that DNA ligase activity has been observed at such high salt concentrations.

As part of a separate study motivated by the discovery of a split NAD^+^-dependent DNA ligase encoded by bacteriophage T5 [5], we also expressed and purified a C-terminally truncated form of the LigN protein comprising only domain 1a and the adenylation and OB fold domains (amino acids 1–417, see Figure 1A). This protein (LigN-ΔC) was expressed in soluble form and could be purified to apparent homogeneity on Ni-NTA agarose (Figure 1E). Incubation of the LigN-ΔC protein with [^32^P]NAD^+ ^resulted in formation of an enzyme-adenylate complex at similar efficiency to the full-length LigN protein (Figure 1F). That the C-terminal region of the protein encompassing the ZnF, HhH and BRCT domains is not required for formation of the enzyme-adenylate complex is consistent with results obtained with other NAD^+^-dependent DNA ligase enzymes of eubacterial and viral origin [3,20-22]. However, despite being efficiently adenylated, the LigN-ΔC protein was inactive in ligase assays using the λ *Bst*EII substrate (Figure 1G). This result indicates that the region of the protein encompassing the ZnF, HhH and BRCT domains plays an important role in overall LigN function in one or more steps subsequent to enzyme adenylation. Again, similar results have been seen with other NAD^+^-dependent DNA ligases [3,20-22]. Unfortunately, the C-terminal part of LigN comprising the ZnF, HhH and BRCT domains could not be stably expressed in *E.coli*, either alone or in combination with LigN-ΔC, and so prevented our attempts to reconstitute enzyme activity from two separate polypeptides (data not shown).

### LigN is active at high salt concentrations

**Figure 2:**
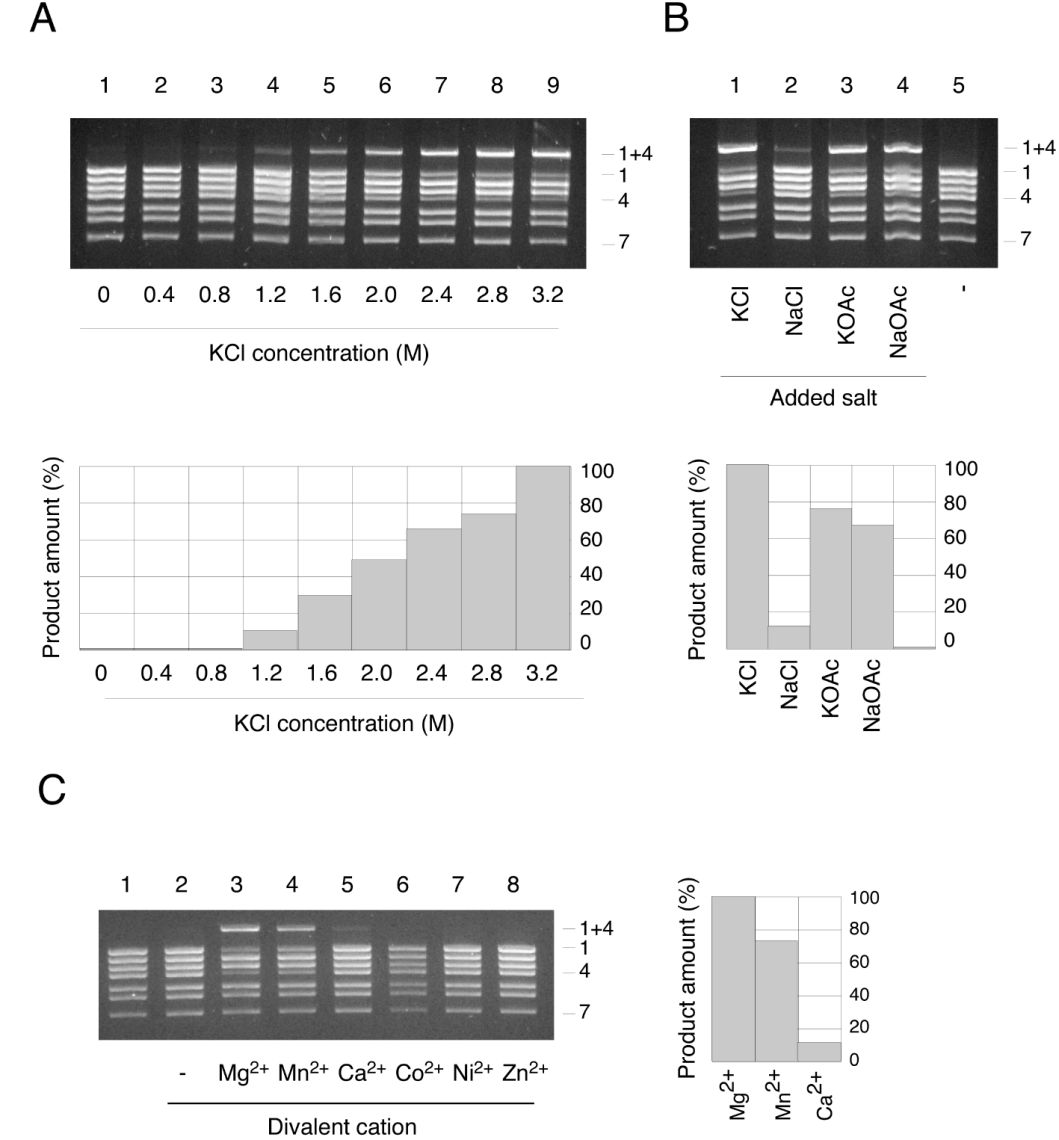
**Salt requirements for LigN activity**. A. DNA ligase activity assays performed under standard conditions (as described in the Methods) at increasing KCl concentrations, from 0 to 3.2 M in 400 mM steps (lanes 1–9). Quantitation of the data is shown below, with the product quantity obtained in 3.2 M KCl (lane 9) indicated as 100%. B. DNA ligase activity assays performed as described in Methods with LigN and 3.2 M KCl (lane 1), 3.2 M NaCl (lane 2), 3.2 M potassium acetate (lane 4), 3.2 M sodium acetate (lane 5) or with no added enzyme (lane 5, reaction performed in 3.2 M KCl). Quantitation of the data is shown below, with the product quantity obtained in 3.2 M KCl (lane 1) indicated as 100%. C. DNA ligase activity assays performed under standard conditions (see Methods) with no added enzyme (lane 1, reaction mix contains 10 mM magnesium acetate) or with LigN and no divalent cation (lane 2), 10 mM MgCl_2 _(lane 3), 10 mM MnCl_2 _(lane 4), 10 mM CaCl_2 _(lane 5), 10 mM CoCl_2 _(lane 6), 10 mM NiCl_2 _(lane 7) and 10 mM ZnCl_2 _(lane 8). Quantitation of the data is shown to the right, with the product quantity obtained in 10 mM MgCl_2 _(lane 3) indicated as 100%.

As noted above, the intracellular KCl concentration of *Hfx.volcanii *cells has been determined as 3.6 M [17]. Using the non-isotopic method described above [19], we tested the activity of LigN at KCl concentrations increasing from 0 to 3.2 M at 400 mM intervals. No activity could be detected in the absence of added KCl or in the presence of either 0.4 M or 0.8 M KCl (Figure 2A, lanes 1–3 respectively). LigN activity was first detected at 1.2 M KCl and increased to a maximum at the highest KCl concentration tested, 3.2 M (Figure 2A, lanes 4–9). The quantitative data shown in Figure 2A might suggest that LigN activity would be higher still at KCl concentrations above 3.2 M but for technical reasons this could not be tested.

To investigate whether KCl specifically was required for LigN activity, similar assays were performed using 3.2 M NaCl, 3.2 M potassium acetate and 3.2 M sodium acetate. The results are shown in Figure 2B. LigN activity was highest with 3.2 M KCl (lane 1), reduced with 3.2 M potassium acetate and 3.2 M sodium acetate (lanes 3 and 4 respectively) and greatly reduced with 3.2 M NaCl (lane 2).

### Additional properties of the LigN enzyme

A general feature of the NAD^+^-dependent ligases is a requirement for a divalent cation for catalysis [2]. Figure 2C shows that this is also true of LigN, since in the absence of a divalent cation, the enzyme is inactive on the λ DNA substrate (Figure 2C, lane 2). The requirement for a divalent cation can be satisfied by 10 mM magnesium or manganese (lanes 3 and 4, respectively), to a much lesser extent by calcium (lane 5) but not by cobalt, nickel or zinc (lanes 6–8).

**Figure 3:**
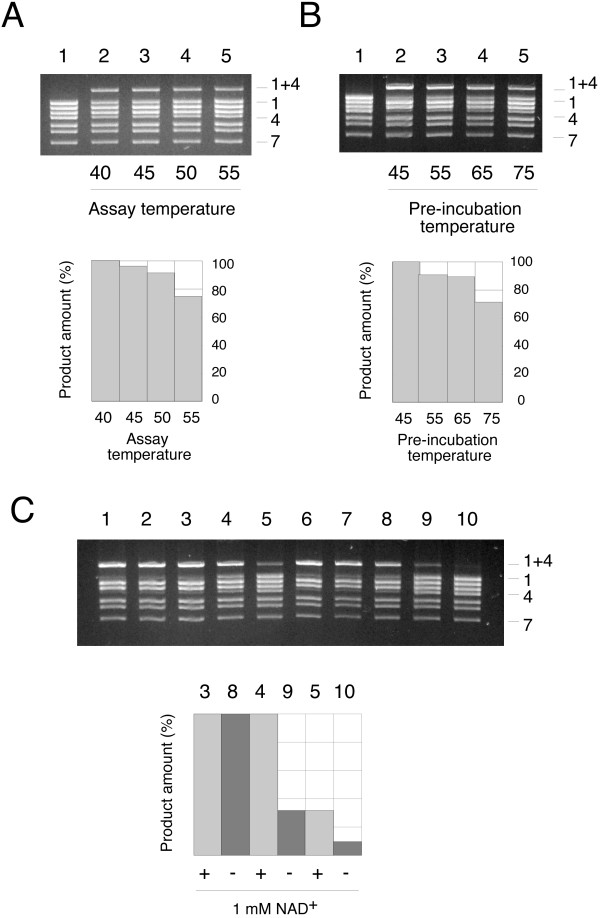
**Additional properties of LigN**. A. LigN activity assays performed at 40°C, 45°C, 50°C and 55°C as indicated in the Methods except only 0.128 pmol of LigN protein (0.2 μg) was used per 20 μl assay. Quantitation of the data is shown to below, with the quantity of product obtained at 40°C indicated as 100%. B. Assays performed under standard conditions (45°C, as indicated in the Methods) following pre-incubation of the LigN protein for 30 minutes at 45°C, 55°C, 65°C or 75°C (lanes 2–5 respectively), or with no LigN protein added (lane 1). Quantitation of the data is shown below, with the quantity of product obtained following preincubation at 45°C indicated as 100%. C. LigN activity assays (20 μl) in the presence (lanes 1–5) or absence (lanes 6–10) of 1 mM NAD^+ ^with 0.64 pmol LigN (lanes 1, 6), 0.128 pmol (lanes 2, 7), 25.6 fmol (lanes 3, 8), 5.12 fmol (lanes 4, 9) and 1.024 fmol (lanes 5, 10) per reaction. The reaction was incubated at 45°C for 6 hours. Quantitation of the data in lanes 3–5 (with NAD^+^) and 8–10 (no added NAD^+^) is shown below, with the product quantities obtained lanes 3 and 8 indicated as 100% for the appropriate datasets.

We also examined temperature and pH dependence for LigN activity in 3.2 M KCl. Figure 3A shows the results of assaying LigN activity at a range of temperatures, from 40 to 55°C, while Figure 3B shows the activity of LigN protein that had been preincubated at 45°C, 55°C, 65°C or 75°C for 30 minutes prior to assaying ligase activity at 45°C. The LigN protein was active over a range of temperatures up to 55°C, significantly higher than the optimum growth temperature of the organism (45°C). Preincubation at temperatures above 45°C resulted in some loss of activity but even after 30 minutes at 75°C, the amount of product formed was reduced by only 40% compared to enzyme that had been preincubated at 45°C. Thus, LigN is a relatively thermostable enzyme. We also found that activity of the enzyme was essentially unchanged over a pH range from 6.8 to 8.8 (data not shown).

To confirm that LigN was NAD^+^-dependent, assays were performed in the absence of the cofactor. We found that the LigN activity was dependent upon the addition of NAD^+^, as expected, but only when the enzyme was present at concentrations significantly lower than the substrate concentration (Figure 3C, lanes 5 and 10). When enzyme concentration equalled or exceeded the substrate concentration (lanes 1–4, 6–9), it was not necessary to add additional NAD^+ ^for efficient ligation, presumably because a significant proportion of the enzyme purified from *E.coli *is in the adenylated state, as observed with other purified recombinant DNA ligases, and one round of nick-sealing is possible.

## Discussion

Since their discovery in the late 1960's [23-27], DNA ligases have been identified and biochemically characterised from a wide variety of cellular organisms and viruses from all three domains of life [1,28]. In this paper we describe the first biochemical analysis of a DNA ligase, LigN, from an extreme halophilic microorganism, the euryarchaeon *Haloferax volcanii*. First isolated from the Dead Sea [8], *Hfx.volcanii *can be easily grown in the laboratory in medium containing 2.5 M NaCl and has attracted attention as a model system for genetic studies of archaeal cell function [29]. By using available genetic tools, we previously showed that LigN was non-essential for *Hfx.volcanii *cell viability but that the enzyme shared an essential function with the archetypal archaeal ATP-dependent ligase LigA [9]. By analogy with other systems, this essential function is most likely to be ligation of the Okazaki fragments generated during chromosome replication.

In common with other haloarchaeal organisms [11,12], *Hfx.volcanii *cells balance the high salt of the extracellular environment by accumulating high intracellular concentrations of potassium chloride, estimated at up to 3.6 M in the case of *Hfx.volcanii *[17]. The success of this strategy obviously requires that *Hfx.volcanii *proteins are active at such extreme salt concentrations. We show here that the *Hfx.volcanii *NAD^+^-dependent DNA ligase family member LigN is inactive in the absence of KCl and displays increasing activity at least up to 3.2 M (Figure 2A), close to the measured intracellular KCl concentration [17]. Using the data from the experiment shown in Figure 3C, it was possible to estimate the specific activity of the recombinant LigN enzyme: in reactions containing 1 mM NAD^+^, 1 fmol of LigN was capable of ligating 9.5 fmol of substrate 5' termini in 300 minutes at 45°C. High activity was also seen when potassium acetate or sodium acetate was substituted for KCl, but not when NaCl was used (Figure 2B), and was shown also to be dependent on the presence of a divalent cation (either magnesium or manganese, Figure 2C) and NAD^+ ^(Figure 3C).

Mutation of the presumptive adenylated lysine, lysine 139, prevented adenylation of the enzyme and subsequent ligation activity (Figure 1C, 1D) while removal of the zinc-binding, helix-hairpin-helix and BRCT domains did not affect adenylation but did prevent ligation (Figure 1F, 1G). Adenylation of the enzyme was also prevented when aspartate 141 was mutated to alanine (Figure 1C), suggesting that the structural change that results from loss of aspartate 141 is sufficient to prevent either NAD^+ ^binding or the adenyltransferase reaction from taking place.

**Figure 4:**
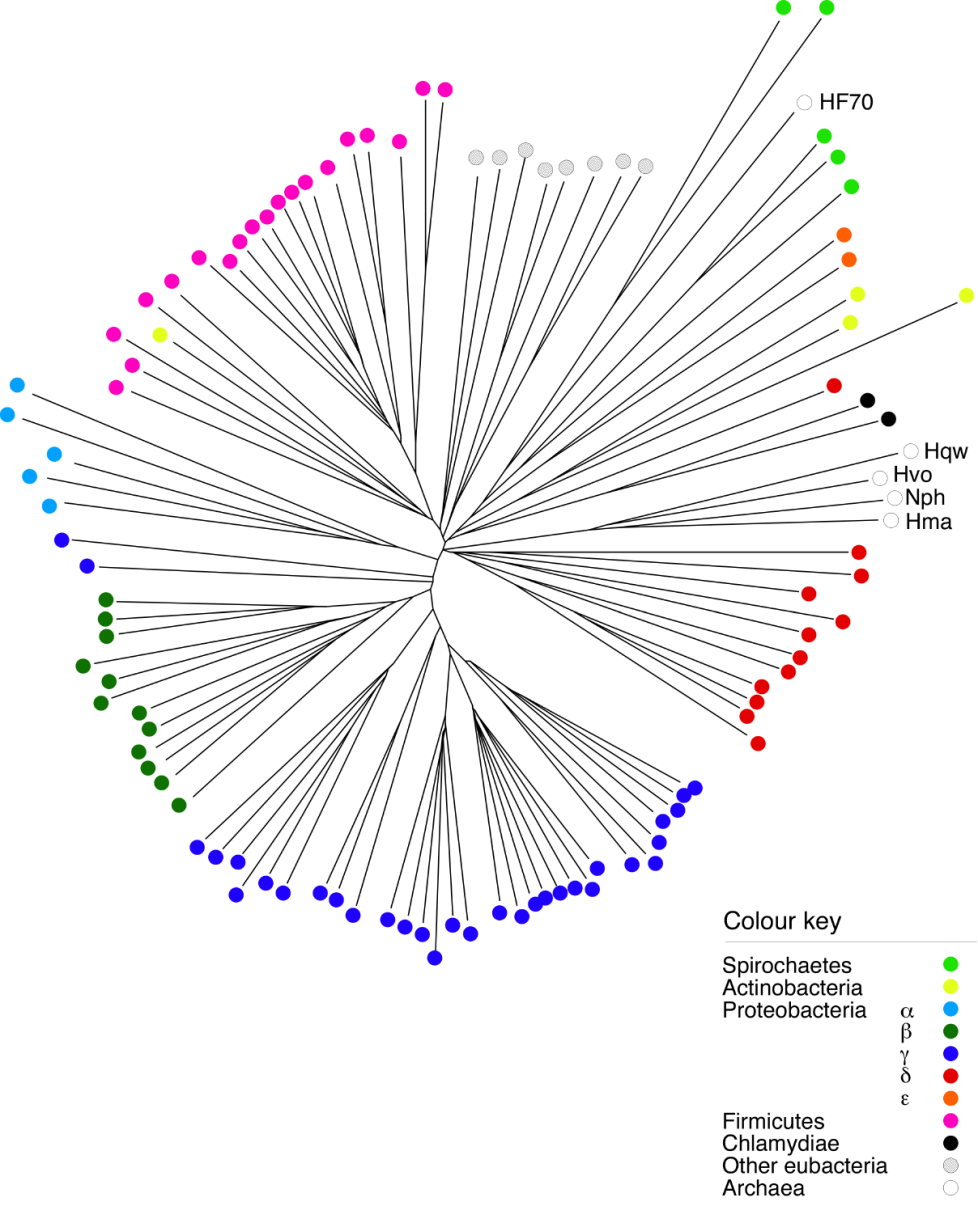
**Phylogenetic analysis of archaeal and eubacterial NAD^+^-dependent DNA ligases**. Phylogenetic tree showing the evolutionary relationship of 106 eubacterial and archaeal NAD^+^-dependent DNA ligase proteins. The tree was generated using TreeView X [39] from sequence alignment data generated using Clustal X [40]. For clarity, only the five proteins of archaeal origin are indicated by name, as follows: *Hvo *(*Haloferax volcanii*), *Hma *(*Haloarcula marismortui*),*Nph *(*Natronomonas pharaosis*), *Hqw *(*Haloquadratum walsbyi *HQ2659A protein) and *HF70 *(*uncultured marine archaeon HF70_B12*). The colour key is shown bottom right. The category labelled *Other eubacteria *includes representatives of the *Chloroflexi*, *Bacteroidetes *and *Deinococci *groups, amongst others. A full listing of the identities and database accession numbers of the proteins used to construct the tree can be obtained from the authors on request.

In an earlier study, we investigated the evolutionary origins of the gene encoding the LigN protein using a dataset of ~80 eubacterial NAD^+^-dependent ligases [9]. Since that analysis was published, two additional archaeal NAD^+^-dependent ligases have been identified by genomic and metagenomic sequencing projects. The first of these is encoded by *Haloquadratum walsbyi*[30]. This protein, which has been designated HQ3698A [31], is closely related to previously-identified haloarchaeal LigN proteins at the primary sequence level (Table 1). Including the sequence of the *H.walsbyi *protein in phylogenetic analysis (Figure 4) does not alter the conclusion that the gene encoding LigN was most likely acquired by a common ancestor of the four haloarchaeal species from a δ-proteobacterial organism [9].

The second potential new NAD^+^-dependent ligase is encoded by a fosmid clone (designated HF70_B12) from an uncultured marine archaeon isolated from a depth of 70 m in the North Pacific [32]. The fosmid carries genes encoding archaeal-type RNA polymerase and ribosome subunits, amongst others; the sequences of these protein are suggestive of a euryarchaeal origin for the fosmid [32]. The overall predicted domain structure of the HF70_B12 encoded enzyme (referred to here as the HF70 ligase) is similar to that of other NAD^+^-dependent ligases including LigN (see Figure 1A) and all the residues predicted to be required for catalysis are present in its primary sequence (data not shown). However, the HF70 ligase is only distantly related to the haloarchaeal enzymes (Table 1). Instead, phylogenetic analysis indicates that the HF70 enzyme is most closely related (typical pairwise protein sequence identities of ~45%) to NAD^+^-dependent ligases from spirochaete eubacteria, represented in the database by two species of each of the genera *Borrelia *and *Treponema *and a single species of the genus *Leptospira *(Figure 4). Thus, the gene encoding the HF70 was almost certainly acquired by an LGT event distinct from that that introduced the gene encoding LigN to the haloarchaea.

Beneficial lateral gene transfer presumably requires that the donor and recipient of the transferred information occupy the same environmental niche. This is an obvious requirement in the case of the halophilic microbes whose proteins are highly salt-adapted. However, many of the sequenced archaeal genomes are from species (such as extreme hyperthermophiles) that do not appear to share their extreme habitats with eubacterial organisms, significantly restricting the opportunities for cross-domain gene transfer. In contrast, marine ecosystems contain an abundance of both eubacterial and archaeal organisms [32]. With this in mind, it seems highly likely that additional cases of LGT of NAD^+^-dependent DNA ligases will be found in marine archaea in the future.

**Figure 5:**
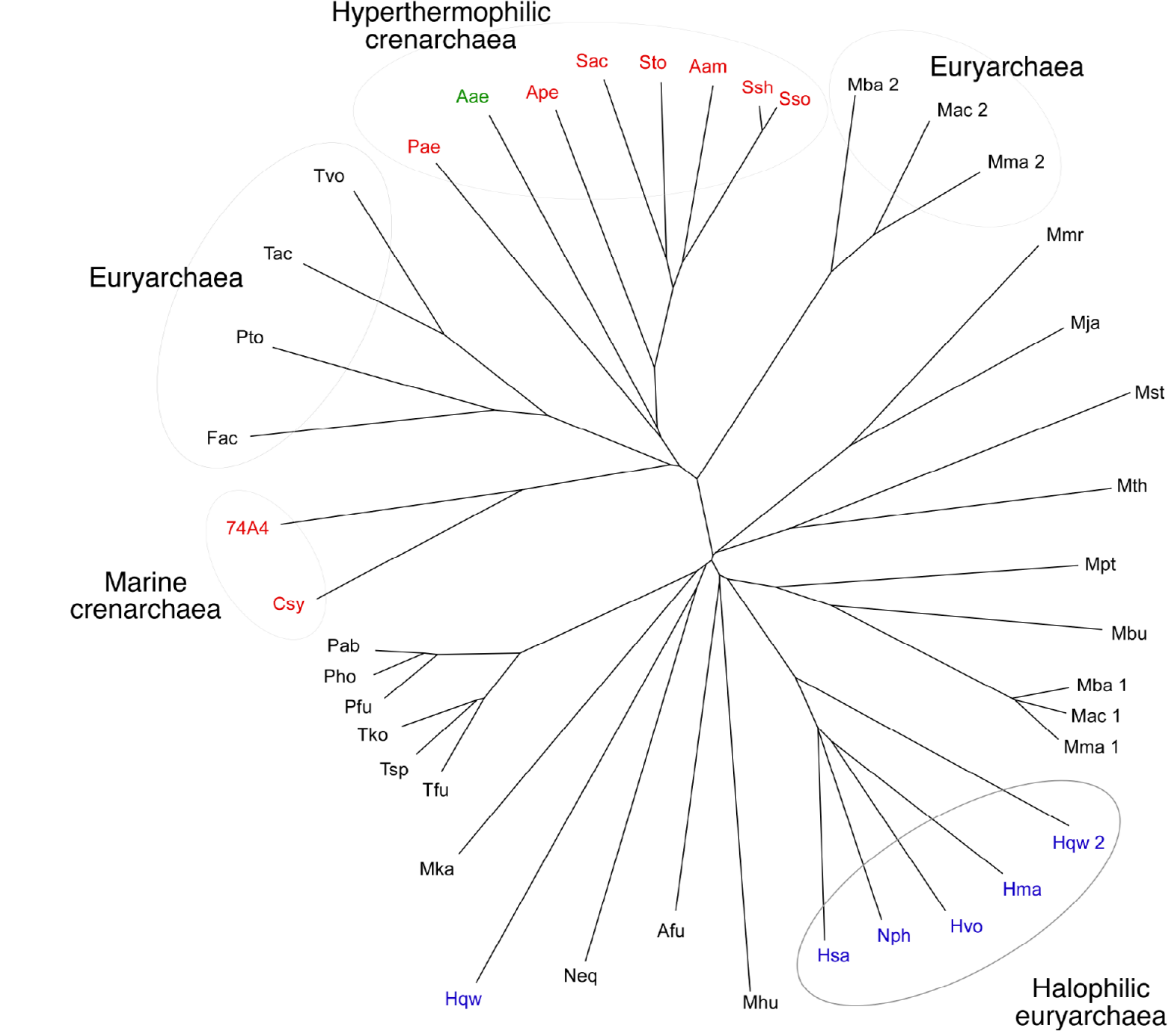
**Phylogenetic analysis of archaeal ATP-dependent DNA ligases**. Phylogenetic tree showing the evolutionary relationship of 41 archaeal ATP-dependent DNA ligase proteins together with the closely-related enzyme encoded by the eubacterium *Aquifex aeolicus*. Crenarchaeal species are shown in red, whereas halophilic euryarchaeal species (such as *Hfx.volcanii*) are shown in blue. The *Aquifex *enzyme (*Aae*, indicated in green) can be seen clustering with the hyperthermophilic (non-marine) crenarchaeal species shown at the top of the figure. Archaeal species abbreviations: *Hvo *(*Haloferax volcanii*), *Aam *(*Acidianus ambivalens*), *Afu *(Archaeoglobus fulgidus), *Ape *(*Aeropyrum pernix*), *Csy *(*Cenarcheon symbiosum*), *Hma *(*Haloarcula marismortui*),*Fac (Ferroplasma acidarmanus), Nph *(*Natronomonas pharaosis*), *Hqw *(*Haloquadratum walsbyi*), *Has *(*Halobacterium salinarum*), *Mac *(*Methanosarcina acetivorans*), *Mba *(*Methanosarcina barkeri*), *Mbu *(*Methanococcoides burtonii*), *Mhu *(*Methanospirillum hungatei*), *Mja *(*Methanocaldococcus jannaschii*), *Mka *(*Methanopyrus kandleri*), *Mma *(*Methanosarcina mazei*), *Mmr *(*Methanococcus maripaludis*), *Mpt *(*Methanosaeta thermophila*), *Mst *(*Methanosphaera stadtmanae*), *Mth *(*Methanothermobacter thermautotrophicus*), *Neq *(*Nanoarchaeum equitans*), *Pab *(*Pyrococcus abyssi*), *Pae *(*Pyrobaculum aerophilum*), *Pfu *(*Pyrococcus furiosus*), *Pho *(*Pyrococcus horikoshii*), *Pto *(*Picrophilus torridus*), *Sac *(*Sulfolobus acidocaldarius*), *Ssh *(*Sulfolobus shibatae*), *Sso *(Sulfolobus solfataricus), *Sto *(*Sulfolobus tokodaii*), *Tac *(*Thermoplasma acidophilum*), *Tfu *(*Thermococcus fumicolans*), *Tko *(*Thermococcus kodakarensis*), *Tsp *(*Thermococcus sp*. NA1), Tvo (*Thermoplasma volcanium*), *74A4 *(*uncultured crenarchaeote 74A4*). The tree was generated using TreeView X [39] from sequence alignment data generated using Clustal X [40]. A full listing of the database accession numbers of the proteins used to construct the tree can be obtained from the authors on request.

To complement our phylogenetic analysis of the NAD^+^-dependent enzymes, we undertook a related investigation of the ATP-dependent DNA ligases encoded by archaeal organisms. A total of 41 proteins were identified from 37 species by database searching, including one enzyme encoded by a fosmid (designated 74A4) derived from an uncultured marine crenarchaeon [33] and one encoded by a fosmid (C03G11) derived from *Cenarchaeum symbiosum*, an uncultured crenarchaeon that lives in a highly specific symbiotic association with a marine sponge [34]. Figure 5 shows a phylogenetic tree based on multiple sequence alignments of the 41 proteins. A single eubacterial ATP-dependent DNA ligase enzyme (from *Aquifex aeolicus*) is also included, as discussed below.

Several features of this tree of worthy of comment. Firstly, there is no clear branching of the tree that separates enzymes from euryarchaeal and crenarchaeal organisms. Four enzymes, from the euryarchaeal species *T.volcanium*, *T.acidophilum*, *P.torridus *and *F.acidarmanus*, appear more closely related to crenarchaeal rather than euryarcheal enzymes (Figure 5). It is possible that a common ancestor of these four species acquired an ATP^+^-dependent ligase from a crenarchaeal organism and then proceeded to lose the gene encoding its own euryarchaeal-type ATP^+^-dependent DNA ligase. Secondly, four euryarchaeal species have the capacity to encode two ATP-dependent DNA ligase enzymes: the newly-sequenced haloarchaeon *H.walsbyi *and three members of the genus *Methanosarcina*. The former is unique amongst sequenced archaea in having the ability to encode three distinct DNA ligase enzymes: two ATP-dependent enzymes (HQ2327A and HQ2659A, see HaloLex [31] database) and one eubacterial-type NAD^+^-dependent enzyme (HQ3698A, discussed above). Phylogenetic analysis indicates that one of the two *H.walsbyi *ATP-dependent enzymes (HQ2659A, indicated as Hqw 1 in Figure 5) is most closely related to ATP-dependent ligase enzymes from the other sequenced haloarchaeal organisms (see Table 2), suggesting that this enzyme is native to the organism, while the other (HQ2327A, indicated as Hqw 2) is most similar to that of the hyperthermophilic obligate symbiont *Nanoarchaeum equitans *(Figure 5), suggesting an LGT origin for the corresponding gene. Similarly, one of the two enzymes encoded by each of the three *Methanosarcina *species (designated Mba 1, Mac 1 and Mma 1 in Figure 5) is closely related to the sole ATP-dependent ligase seen in the related *Methanococcoides *and *Methanosaeta *species, while the other (designated Mba 2, Mac 2 and Mma 2 in Figure 5) is more closely related to the crenarchaeal enzymes. Again, LGT is likely to have played a major role in the transfer of ligase genes from organism to organism within the archaea and between the archaea and the eubacteria.

Database searching reveals genes encoding putative ATP-dependent DNA ligases are present in many eubacterial species [35]. Biochemical and genetic analysis has demonstrated that the encoded proteins can have important functions in the repair of DNA damage [36] as well as in the mechanism of genome circularisation employed by certain bacteriophage [37]. While a detailed analysis of the evolutionary origins of the members of this diverse group of enzymes lies outside the scope of this report, the enzyme encoded by the hyperthermophilic eubacterium *Aquifex aeolicus *is worthy of comment. This DNA ligase is most closely related to enzymes encoded by hyperthermophilic crenarchaeal organisms such as *Acidianus ambivalens*, *Sulfolobus solfataricus, Aeropyrum pernix *and *Pyrobaculum aerophilum *(Figure 5). In each case, the protein sequence identity is ~50% (Table 3), suggesting that acquisition of the gene by *A.aeolicus *by LGT was a relatively recent event.

## Conclusion

The results reported here represent the first biochemical analysis of a DNA ligase enzyme derived from an extremely halophilic organism. *Hfx.volcanii *LigN enzyme activity is completely dependent upon addition of salt and is optimal at concentrations of 3.2 M or higher, consistent with the measured intracellular KCl concentration of *Hfx.volcanii *cells. LigN has great potential both as a novel tool for biotechnology and as a model enzyme for studying adaptation of proteins to high intracellular salt levels.

## Methods

### Bacterial strains and media

For routine cloning procedures, *E.coli *XL1-Blue (Stratagene) was used. Recombinant protein expression was carried out using *E.coli *Rosetta 2 (DE3) [pLysS] (Novagen). LB medium [38] was used throughout, with appropriate antibiotic supplements to select for plasmids. Antibiotics were purchased from Sigma-Aldrich.

### Molecular biological and biochemical reagents

Restriction enzymes, T4 DNA ligase, *Taq *DNA ligase buffer and *Bst*EII-digested λ DNA were obtained from New England Biolabs, [^32^P]NAD^+ ^from GE Healthcare, and oligonucleotides from DNA Technology A/S. All other chemicals were obtained from Sigma-Aldrich. Amplification of *Hfx.volcanii *DNA was accomplished using the GC-rich PCR system (Roche Applied Science). DNA sequencing was performed in-house using DYEnamic ET dye terminator reagent (GC Biotech) and an ABI 310 Gene Analyzer. Plasmid DNA was prepared using either of the QIAprep Spin Miniprep or QIAfilter Plasmid Midi kits (Qiagen). DNA fragment purification was accomplished using various MinElute DNA purification kits (Qiagen).

### LigN expression plasmids

Plasmid pET47b-HfxLigN has been described previously [9]. For expression of LigN-K139A and LigN-D141A, the *ligN*^+ ^insert in this plasmid was modified using the PCR overlap extension mutagenesis technique [38]. With pET47b-HfxLigN as the template, PCR products were generated using oligonucleotide pairs K1 (specifically oligos PET47B-5SEQ and HVOLIGN-KR, see Table 4) and D1 (PET47B-5SEQ and HVOLIGN-DR), combined with the products generated using pairs K2 (HVOLIGN-PCR-1R and HVOLIGN-KF) and D2 (HVOLIGN-PCR-1R and HVOLIGN-DF) respectively, and subjected to further amplification using the flanking primers PET47B-5SEQ and HVOLIGN-PCR-1R only. The final ~950 bp PCR product was then restricted with *Xma*I and *Bgl*I and the 500 bp fragment containing the mutated region re-cloned into pET47b-HfxLigN from which the corresponding non-mutated *Xma*I- *Bgl*I region had been removed. The resulting plasmids (pET47b-HfxLigN-K139A and pET47b-HfxLigN-D141A) were sequenced to confirm the success of the mutagenesis procedure and the absence of unwanted sequence changes.

For expression of LigN-ΔC, a ~1300 bp region of the *ligN*^+ ^gene, corresponding to sequences encoding amino acids 1–417, was amplified by PCR using oligonucleotides HVOLIGN-N-5BAM and HVOLIGN-N-3H (see Table 4), restricted with *Bam*HI and *Hind*III, and cloned into plasmid pETDuet-1 (Novagen) that had been digested with the same enzymes. Again, the resulting plasmid (pETDuet-1-HfxLigN-ΔC) was sequenced to confirm the absence of unwanted sequence changes.

### Expression and purification of recombinant LigN proteins

1 litre cultures of *E.coli *Rosetta 2 (DE3) [pLysS] (Novagen) cells transformed with either pET47b-HfxLigN plasmids or pETDuet-1-HfxLigN-ΔC were grown with shaking at 37°C in LB medium supplemented with chloramphenicol (34 μg/ml) and either kanamycin (25 μg/ml, for pET47b plasmids) or ampicillin (100 μg/ml, for pETDuet-1-HfxLigN-ΔC) to an OD_600nm _of 0.6, at which point IPTG was added to a final concentration of 1 mM and growth continued for a further 4 hours at 37°C. Cells were then harvested by centrifugation in a pre-chilled Sorvall GS-3 rotor (4°C, 6000 g, 10 minutes) and pellets stored at -20°C overnight. The cell pellets were then thawed at room temperature for 30 minutes, resuspended in 20 ml of buffer 1 (20 mM NaH_2_PO_4_, 20 mM imidazole, 2.0 M KCl, 0.1% Igepal CA-630, pH 8.0) and lysed by sonication (on ice, using six 10 second pulses at 10% power output with 15 second rest periods on a 200 W, 20 kHz Bandelin Sonopuls HD2200 sonicator). The lysate was then centrifuged in a Sorvall SS-34 rotor (4°C, 27000 g, 10 minutes × 4) and the cleared supernatant applied to a 1.5 ml NiNTA Superflow column (Qiagen) according to the manufacturer's instructions. The column was washed five times with 10 ml of buffer 2 (20 mM NaH_2_PO_4_, 2.0 M KCl, pH 8.0) and the bound proteins then eluted with five 2 ml aliquots of ice-cold buffer 3 (20 mM NaH_2_PO_4_, 500 mM imidazole, 2.0 M KCl, pH 8.0). 500 μl fractions were collected and analysed by SDS-PAGE. Peak fractions (generally 3–4 fractions were selected) were pooled and dialysed overnight against 2 litres of buffer 2 at room temperature with stirring. Protein concentrations were determined using the BCA assay kit (Pierce). Purified proteins were then stored at 4°C. The yield from 1 litre of culture was ~8 mg. The purified proteins appeared essentially homogeneous by SDS-PAGE (see Figures 1B, 1E).

### Adenyltransferase activity assays

Reaction mixtures (20 μl) contained 20 mM NaH_2_PO_4_, 10 mM MgCl_2_, 2.0 M KCl, pH 8.0, 1 μM [^32^P] NAD^+ ^(nicotinamide adenine [adenylate-^32^P] dinucleotide, from GE Healthcare) and 10 pmol of ligase protein. After incubation at room temperature for 15 minutes, the reaction was desalted using a Zeba desalt spin column (Pierce) according to the manufacturer's instructions. 20 μl of 2× SDS PAGE sample buffer was then added and the reaction heated to 95°C for 5 minutes prior to 10% SDS-PAGE. Dried gels were then exposed to BioMax XAR film (Kodak) at -20°C. Quantitation was achieved using a Cyclone Storage Phosphor System and OptiQuant software (Perkin-Elmer) according to the manufacturer's instructions.

### Ligase assays

DNA ligase assays were performed according to the method of Muerhoff et al. [19] with minor modifications. Unless otherwise stated, standard reactions (20 μl) contained 6.4 pmol (0.5 μg) of LigN in 20 mM Tris-HCl (pH 7.6 at 25°C), 3.2 M KCl, 25 mM potassium acetate, 10 mM magnesium acetate, 10 mM DTT, 1 mM NAD^+^, 0.1% Triton X100 and 500 ng of *Bst*EII-digested λ DNA (New England Biolabs) and were performed at 45°C for 10 minutes. Reactions were stopped by addition of 4.0 μl of 50 mM EDTA. The samples were then desalted using the MinElute reaction clean-up kit (Qiagen), according to the manufacturer's instructions, eluted in 22 μl of EB buffer (10 mM Tris pH 8.0), heated to 65°C for 5 minutes and chilled on ice for 5 minutes prior to loading onto a 1% agarose gel prepared in 1 × TAE buffer. The gel was run at 120 V for 120 minutes. Both the gel and the buffer contained 175 ng/ml ethidium bromide. Band intensities were determined by measuring UV fluorescence using 1D Image Analysis software (Kodak) with exposure times maximised to give high band intensity without pixel saturation. Activity values were calculated by measuring the net intensity of the product band (indicated as 1+4 in the figures) relative to a non-substrate fragment (fragment 7).

## Authors' contributions

LP performed most of the biochemical work and prepared some of the expression plasmids. SM conceived of the study, prepared the remaining plasmids, performed some of the biochemical experiments, analysed the data and wrote the manuscript. Both authors read and approved the final manuscript.

## Figures and Tables

**Table 1:** Pairwise primary sequence identity (%) between haloarchaeal NAD^+^-dependent DNA ligases.

	*Hvo*	*Hma*	*Nph*	*Hqw*	*HF70*
*Hvo*	100	62.1	55.4	60.1	32.1
*Hma*	62.1	100	61.7	58.2	32.3
*Nph*	55.4	61.7	100	54.0	31.8
*Hqw*	60.1	58.2	54.0	100	31.9
*HF70*	32.1	32.3	31.8	31.9	100

Abbreviations: *Hvo *(*Haloferax volcanii*), *Hma *(*Haloarcula marismortui*),*Nph *(*Natronomonas pharaosis*), *Hqw *(*Haloquadratum walsbyi *HQ2659A protein) and *HF70 *(*uncultured marine archaeon HF70_B12*). Sequence identities were calculated using default settings with the SIM alignment tool [41]

**Table 2:** Pairwise primary sequence identity (%) between haloarchaeal ATP-dependent DNA ligases.

	*Hvo*	*Hma*	*Nph*	*Hsa*	*Hqw 1*	*Hqw 2*
*Hvo*	100	65.4	66.3	65.8	53.8	33.1
*Hma*	65.4	100	67.5	64.6	50.3	35.6
*Nph*	66.3	67.5	100	69.5	55.2	37.3
*Has*	65.8	64.6	69.5	100	51.9	36.3
*Hqw1*	53.8	50.3	55.2	51.9	100	31.2
*Hqw2*	33.1	35.6	37.3	36.3	31.2	100

Abbreviations: *Hvo *(*Haloferax volcanii*), *Hma *(*Haloarcula marismortui*),*Nph *(*Natronomonas pharaosis*), *Hsa *(*Halobacterium salinarum*), *Hqw 1 *(*Haloquadratum walsbyi *HQ2659A protein) and *Hqw 2 *(*H.walsbyi *HQ2327A protein). Sequence identities were calculated using default settings with the SIM alignment [41].

**Table 3:** Pairwise primary sequence identity (%) between crenarchaeal ATP-dependent DNA ligases and that of the eubacterium *Aquifex aeolicus*.

	*Aam*	*Ssh*	*Ape*	*Pae*	*Aae*	*74A4*
*Aam*	100	74.8	59.3	49.0	50.8	42.4
*Ssh*	74.8	100	59.1	48.1	49.4	42.4
*Ape*	59.3	59.1	100	50.8	48.1	41.0
*Pae*	49.0	48.1	50.8	100	47.5	44.3
*Aae*	50.8	49.4	48.1	47.5	100	42.9
*74A4*	42.4	42.4	41.0	44.3	42.9	100

Abbreviations: *Aam *(*Acidianus ambivalens*, formerly *Desulfurolobus ambivalens*), *Ssh *(*Sulfolobus shibatae*),*Ape *(*Aeropyrum pernix*), *Pae *(*Pyrobaculum aerophilum*), *Aae *(*Aquifex aeolicus*), and *74A4 *(uncultured marine crenarchaeon *74A4*). Sequence identities were calculated using default settings with the SIM alignment tool [41].

**Table 4:** Oligonucleotide primers used in this study.

Oligonucleotide	Sequence (5'-3')
HVOLIGN-KF	GATTACGTCTGCGAACCCGCGTTCGACGGCCTCTCGGTC
HVOLIGN-KR	GACCGAGAGGCCGTCGAACGCGGGTTCGCAGACGTAATC
HVOLIGN-DF	GTCTGCGAACCCAAGTTCGCCGGCCTCTCGGTCGAAGTC
HVOLIGN-DR	GACTTCGACCGAGAGGCCGGCGAACTTGGGTTCGCAGAC
HVOLIGN-PCR-1R	CGGTGACGCGGAGACCCCACTCGC
PET47B-5SEQ	GCCTAGGTTAATTAAGCCTCGAGA
HVOLIGN-N-5BAM	GTTGGTTGGTggatccGATGAGCGACGCCGACGTCGACGC
HVOLIGN-N-3H	GTGTGTGTGTaagcttCTACCCGCCGCCGTCTTCGGTGAC

Note: Sequencing primers are not shown but details are available from the authors on request. Restriction sites are shown in lower case.
